# CD34 evaluation of microvasculature in lung adenocarcinoma and its microvascular density predicts postoperative tumor recurrence

**DOI:** 10.3389/pore.2025.1611985

**Published:** 2025-01-20

**Authors:** Zijian Qiu, Jiaji Wu, Guanchao Pang, Xia Xu, Jun Lin, Pingli Wang

**Affiliations:** ^1^ Department of Radiation Oncology, The Quzhou Affiliated Hospital of Wenzhou Medical University, Quzhou People’s Hospital, Quzhou, Zhejiang, China; ^2^ Department of Respiratory Medicine, The Fourth Affiliated Hospital of Zhejiang University School of Medicine, Yiwu, Zhejiang, China; ^3^ Department of Respiratory and Critical Care Medicine, The Second Affiliated Hospital of Zhejiang University School of Medicine, Hangzhou, Zhejiang, China; ^4^ Department of Pathology, The Second Affiliated Hospital of Zhejiang University School of Medicine, Hangzhou, Zhejiang, China; ^5^ Department of Pathology, The Quzhou Affiliated Hospital of Wenzhou Medical University, Quzhou People’s Hospital, Quzhou, Zhejiang, China

**Keywords:** CD34, microvascular density, lung adenocarcinoma, prognosis, tumor recurrence

## Abstract

**Background:**

Angiogenesis is closely associated with tumor growth and metastasis, and microvascular density (MVD) is currently the clinical standard for evaluating tumor angiogenesis. Thus, the detection of intratumoral MVD is of great significance for understanding disease progression and predicting patient prognosis.

**Methods:**

Tumor tissue sections of 238 patients with lung adenocarcinoma (LUAD) who underwent radical surgery were retrospectively analyzed. Immunohistochemical (IHC) staining was carried out using a CD34 polyclonal antibody to determine intratumoral MVD, and the relationship of CD34-MVD with the clinicopathological characteristics and survival time of LUAD patients was analyzed.

**Results:**

CD34-MVD was associated with tumor size, lymph node metastasis, tumor recurrence, and patient survival status; patients with tumor size ≤3 cm (*P* = 0.015), negative for lymph node metastasis (*P* = 0.049), no tumor recurrence (*P* = 0.021), and survival (*P* = 0.042) had higher MVD. Survival analysis suggested that patients with high MVD had higher disease-free survival (log-rank *P* = 0.005) and overall survival (log-rank *P* = 0.004) compared to patients with low MVD. The Cox proportional hazards model showed that a high MVD (*P* = 0.022) reduced the risk of postoperative tumor recurrence in patients with LUAD.

**Conclusion:**

Decreased intratumoral CD34 positive microvessels were associated with tumor development in patients with LUAD. CD34-MVD is an independent risk factor affecting postoperative tumor recurrence in patients with LUAD and can be used as a prognostic indicator for this group of patients.

## Introduction

Lung cancer is currently the most common malignancy with the highest mortality rate worldwide [1]. In China, lung cancer ranks first among all malignancies in terms of incidence and mortality rate [2]. Lung adenocarcinoma (LUAD) is the most common pathological type of lung cancer, accounting for approximately 45% of all cases, and has a significantly higher incidence than other pathological subtypes such as squamous cell, small cell, and large cell carcinomas [3]. Surgical resection is the mainstay of treatment for early to intermediate stage (stage I–IIIA) nonsmall cell lung cancer (NSCLC), but the risk of tumor recurrence and death after radical surgery remains high [4]. Therefore, the search for molecular markers associated with tumor progression or prognosis in lung cancer is of great significance for elucidating tumor pathogenesis and screening patients with poor prognosis [5, 6].

Tumor growth is accompanied by angiogenesis, and antiangiogenic therapy has always been a crucial target in antitumor treatment [7]. The relationship between tumor angiogenesis and the prognosis of tumor patients has also been widely studied and is currently a hot topic of discussion. Microvascular density (MVD) is an indicator used to assess the number of blood vessels within a tumor [8] and plays an important role in predicting the prognosis of patients with tumors [9–11].

CD34 is a pan-endothelial cell marker commonly used to assess tumor vascularity. Although researchers have used CD34 antibodies to label the tumor microvasculature by performing immunohistochemical (IHC) staining of lung cancer tumor tissues and to assess the relationship between MVD, tumor progression, and patient prognosis, the results of different studies have been controversial. A study by Tanaka et al. [12] that included 236 NSCLC patients found that intratumoral CD34-MVD was not associated with tumor stage or five-year survival rate. Similarly, Donnem et al. found that among 335 NSCLC patients, high vs. low CD34-MVD was not associated with a five-year survival rate [13]. In contrast, Kadota et al. found that among 147 NSCLC patients, those with high CD34-MVD had lower survival rates [14]. In a study of 81 patients with NSCLC, Bing et al. showed that an increase in CD34-MVD was associated with tumor progression but not lymph node metastasis; unfortunately, the study did not include a survival analysis [15]. The results of the study by Pomme et al., which included 371 NSCLC patients, were contrary to all the studies above and instead showed that the increase in CD34-MVD was associated with lower TNM stage and better prognosis [16]. Furthermore, all the above studies included NSCLC patients, and none analyzed patients with lung adenocarcinoma separately. Therefore, the relationship between CD34-MVD and tumor progression and prognosis in patients with lung adenocarcinoma remains inconclusive and warrants further investigation.

In this study, IHC staining was performed using CD34 antibodies on tumor tissue sections from 238 LUAD patients who underwent radical surgery to determine intratumoral MVD and to analyze the relationship between CD34-MVD and patient clinicopathological characteristics and survival. Based on the above, we aimed to explore the role of CD34-MVD in tumor development and its prognostic value.

## Materials and methods

### Patients

Patients enrolled in this study were required to meet the following conditions: 1) a definitive pathological diagnosis of LUAD with no history of other tumors; 2) no neoadjuvant treatments, such as chemotherapy and radiotherapy, before surgery; 3) radical surgery for lung cancer to ensure complete tumor resection; 4) postoperative stage within stages I–III, excluding patients with distant metastases; and 5) postoperative tumor tissues formally sampled by the Department of Pathology and used to prepare tissue wax blocks, which were well preserved and usable.

In strict accordance with the criteria above, we screened and enrolled 238 patients who underwent radical lung cancer surgery between 1 January 2011, and 30 December 2015, at the Second Affiliated Hospital of Zhejiang University School of Medicine. The age of these patients ranged from 26 to 87 years, with a mean age of 60.48 years. The distributions of other characteristics are detailed in Table 1.

**TABLE 1:** Relationship between MVD and clinicopathologic characteristics.

Grouping	Number (proportion %)	MVD( $\bar{x}$ ± s)	Difference value	95% CI	T test/F test	
					T/F value	*P* value
Age, years						
≤60	108 (45.4%)	20.16 ± 6.414	−0.235	−1.867∼1.398	−0.283	0.777
>60	130 (54.6%)	20.39 ± 6.323				
Sex						
Female	133 (55.9%)	20.77 ± 6.450	1.091	−0.540∼2.722	1.317	0.189
Male	105 (44.1%)	19.68 ± 6.203				
Smoking						
Never	166 (69.7%)	20.41 ± 6.531	0.410	−1.359∼2.178	0.456	0.649
Have	72 (30.3%)	20.00 ± 5.953				
Tumor Site						
Left lung	106 (44.5%)	19.78 ± 6.123	−0.906	−2.538∼0.725	−1.095	0.275
Right lung	132 (55.5%)	20.68 ± 6.526				
Tumor Differentiation						
Well	59 (24.8%)	21.12 ± 5.986	—	19.56∼22.68	2.055	0.130
Moderately	85 (35.7%)	20.84 ± 6.766		19.38∼22.29		
Poorly	94 (39.5%)	19.27 ± 6.112		18.01∼20.52		
Pathological Subtypes						
Acinar	127 (53.4%)	20.69 ± 6.699	—	19.52∼21.87	2.013	0.078
Lepidic	35 (14.7%)	21.69 ± 5.718		19.72∼23.65		
Micropapillary	9 (3.8%)	21.78 ± 4.919		18.00∼25.56		
Papillary	31 (13.0%)	18.55 ± 5.525		16.52∼20.57		
Solid	18 (7.6%)	20.22 ± 6.967		16.76∼23.69		
Variant	18 (7.6%)	17.00 ± 5.224		14.40∼19.60		
Tumor size						
≤3 cm	131 (55.0%)	21.34 ± 5.887	2.000	0.393∼3.608	2.451	**0.015**
>3 cm	107 (45.0%)	19.34 ± 6.626				
T Stage						
T1-2	186 (78.2%)	20.34 ± 6.224	0.243	−1.724∼2.209	0.243	0.808
T3-4	52 (21.8%)	20.10 ± 6.849				
N Stage						
N0	132 (55.5%)	21.00 ± 6.699	1.604	0.006∼3.201	1.978	**0.049**
N+	106 (44.5%)	19.40 ± 5.801				
TNM Stage						
Stage I	74 (31.1%)	21.54 ± 6.513	—	20.03∼23.05	2.860	0.059
Stage II	73 (30.7%)	19.05 ± 6.506		17.54∼20.57		
Stage III	91 (38.2%)	20.25 ± 5.955		19.01∼21.49		
Tumor Recurrence						
Yes	122 (51.3%)	19.36 ± 5.972	−1.898	−2.325∼−0.290	−2.325	**0.021**
No	116 (48.7%)	21.26 ± 6.615				
Status of Survival						
Death	63 (26.5%)	18.89 ± 5.949	−1.900	−3.726∼−0.073	−2.049	**0.042**
Survival	175 (73.5%)	20.79 ± 6.433				

CI, confidence interval; MVD, microvascular density. Significant *P* values are presented in bold.

### Information acquisition and follow-up

The clinicopathological characteristics of all patients were collected using the hospital information system. These included patient name, sex, age, smoking status, tumor location, tumor size, degree of differentiation, pathological subtype, and TNM stage. Postoperative TNM staging of all patients with LUAD was performed according to the 8th edition of the Union for International Cancer Control staging criteria.

Follow-up was conducted through a combination of accessing the hospital information system (outpatient and inpatient medical records) and telephone consultation. Follow-up information included whether the patients experienced tumor recurrence, whether they survived, and the time to a positive event after radical lung cancer surgery. Disease-free survival (DFS) was calculated as the time from complete resection to tumor recurrence. Overall survival (OS) was calculated as the time from the patient’s diagnosis of LUAD to death.

### Research ethics

This study was approved by the Ethics Committee of the Second Affiliated Hospital of the Zhejiang University School of Medicine (Document batch number 2019-308). Informed consent was waived by our Ethics Committee because of the retrospective nature of our study. We confirm that all methods were performed in accordance with the relevant guidelines and regulations.

### IHC staining

Tumor tissue wax blocks were sliced to obtain tissue sections with a thickness of 3–5 μm.

The tissue slides were deparaffinized and hydrated using xylene, ethanol, and distilled water in turn. Antigen retrieval: Citrate buffer (PH 6.0) was poured into the pressure cooker and heated; After boiling, the slides were added and heated for 2 min under closed conditions; The slides were washed with phosphate-buffered saline (PBS) (PH7.4) after cooling. Quenching endogenous peroxidase activity: Incubate slides with 3% H2O2 solution for 10 min at room temperature; After washing with PBS (PH7.4), serum blocking was performed by adding 3% bovine serum albumin (Haokebio, HK5021, Hangzhou, China) for 30 min at room temperature. Antibody incubation: Primary antibody CD34 (Proteintech, 14486-1-ap, Wuhan, China) prepared with PBS (PH7.4) at a concentration ratio of 1:800 was added and incubated for 90 min. After washing with PBS (PH7.4), ultrasensitive rabbit and mouse universal secondary antibodies (Biolynx, I20012B, Hangzhou, China) was added and incubated for 30 min. Chromogenic reaction: After washing with PBS (PH7.4), diaminobenzidine chromogenic solution (Biolynx, I20012C, Hangzhou, China) was added (diluent and concentrate were prepared at a ratio of 1,000:50) and incubated for 2–5 min. Positive color reactions appeared brownish-yellow, and the color development was terminated by rinsing with pure water. After color development, hematoxylin staining solution was used to stain the nuclei. Finally, the slides were dehydrated and mounted. After microscopic examination, the section images were scanned and captured using a digital pathology slide scanner (KFBIO, KF-PRO-120, Ningbo, China).

### Microvasculature interpretation and MVD counting

The sections were read using digital slice-reading software K-VIEWER (KFBIO, ver1.7.1.1, Ningbo, China). Based on the Weidner correction method [17], any single endothelial cell or cell mass stained by the antibody, whether it formed a lumen, was considered to be a countable microvessel as long as it was clearly demarcated from the surrounding microvasculature, tumor cells, and other connecting tissues. Microvessels within sclerotic areas of the tumor and soft tissues at the tumor margins were not counted. Vessels with smooth muscle walls and lumen diameters greater than eight red blood cells were also excluded. For each specimen, three areas with the highest number of microvessels were selected at low power (×10), that is, “hotspots.” The number of microvessels was counted for each high-power field (HPF, ×40), and the average value was taken as the MVD value. All procedures were performed independently by two experienced pathologists.

### Statistical analysis

Data were analyzed using the statistical package SPSS 25.0 and R version 4.2.1. The *t*-test or F-test was used to analyze the association between MVD and the clinicopathological characteristics. The Kaplan-Meier method was used to calculate the survival rate, and the log-rank test was used to compare differences in survival. Cox proportional hazards regression analysis was used for univariate and multivariate analyses to predict survival. Differences were considered statistically significant at *P* < 0.05.

## Results

### CD34 positive microvessels in LUAD

**FIGURE 1:**
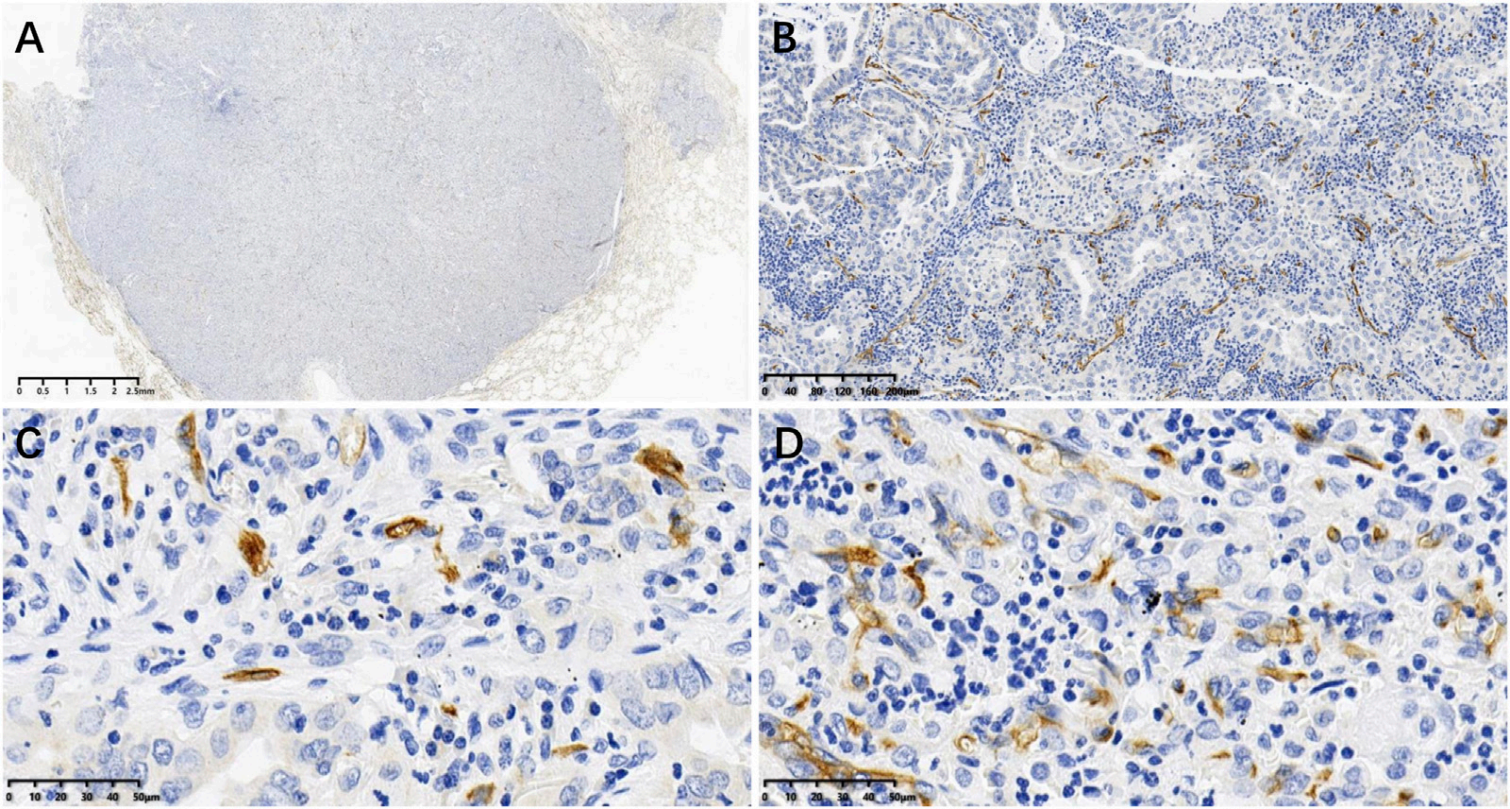
CD34 positive microvessels in LUAD tissues: **(A)** Image acquired by digital pathology scanner; **(B)** CD34 positive microvessels at low power (×10); **(C, D)** CD34 positive microvessels at high power (×40), where **(C)** is low MVD (MVD ≤20 vessels) and D is high MVD (MVD >20 vessels).

CD34 positive microvessels in LUAD is shown in Figure 1. MVD ranged between 8 and 38 vessels per HPF (×40), with a mean of 20.29 ± 6.352 vessels. Using the mean value as a cutoff, patients with an MVD ≤20 were assigned to the low-MCD group and those with an MVD >20 to the high-MVD group.

### Relationship between MVD and clinicopathologic features

When grouped by tumor size, patients in the ≤3 cm group had an MVD of 21.34 ± 5.887, while those in the >3 cm group had an MVD of 19.34 ± 6.626, with the ≤3 cm group showing a higher MVD (T = 2.451, *P* = 0.015). When grouped by N-stage, patients without lymph node metastasis (N0) had an MVD of 21.00 ± 6.699, while those with lymph node metastasis (N+) had an MVD of 19.40 ± 5.801, with the N0 group showing a higher MVD (T = 1.978, *P* = 0.049). When grouped by tumor recurrence, patients in the recurrence group had an MVD of 19.36 ± 5.972, while those in the non-recurrence group had an MVD of 21.26 ± 6.615, and the recurrence group showed a lower MVD (T = −2.325, *P* = 0.021). When grouped by mortality status, patients in the deceased group had an MVD of 18.89 ± 5.949, while those in the non-deceased group had an MVD of 20.79 ± 6.433, with the deceased group showing a lower MVD (T = −2.049, *P* = 0.042).

There was no statistically significant difference in MVD when the patients were grouped by age, sex, smoking status, tumor location, degree of differentiation, T stage, and TNM stage (*P* ≥ 0.05).

### CD34 positive microvessels in different pathological subtypes

**FIGURE 2:**
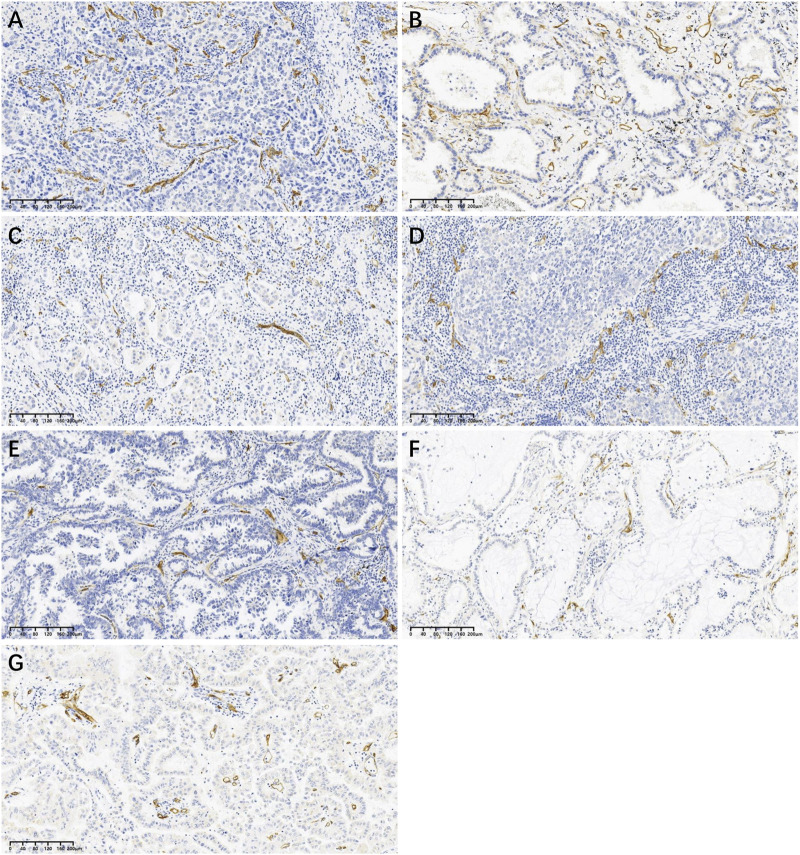
Representative images of CD34 positive microvessels in each pathological subtype at low power (×10): **(A)** Acinar subtype; **(B)** Lepidic subtype; **(C)** Micropapillary subtype; **(D)** Solid subtype; **(E)** Papillary subtype; **(F)** Mucinous adenocarcinoma (one of variant subtypes); **(G)** Enteric adenocarcinoma (one of variant subtypes).

LUAD can be divided according to its growth pattern into five main pathological subtypes: lepidic, papillary, micropapillary, acinar, and solid. Based on the data in Table 1, we found that the MVD was much lower in the papillary subtype and variant subtype. Representative images of CD34 positive microvessels in each pathological subtype are shown in Figure 2.

### Survival analysis

**FIGURE 3:**
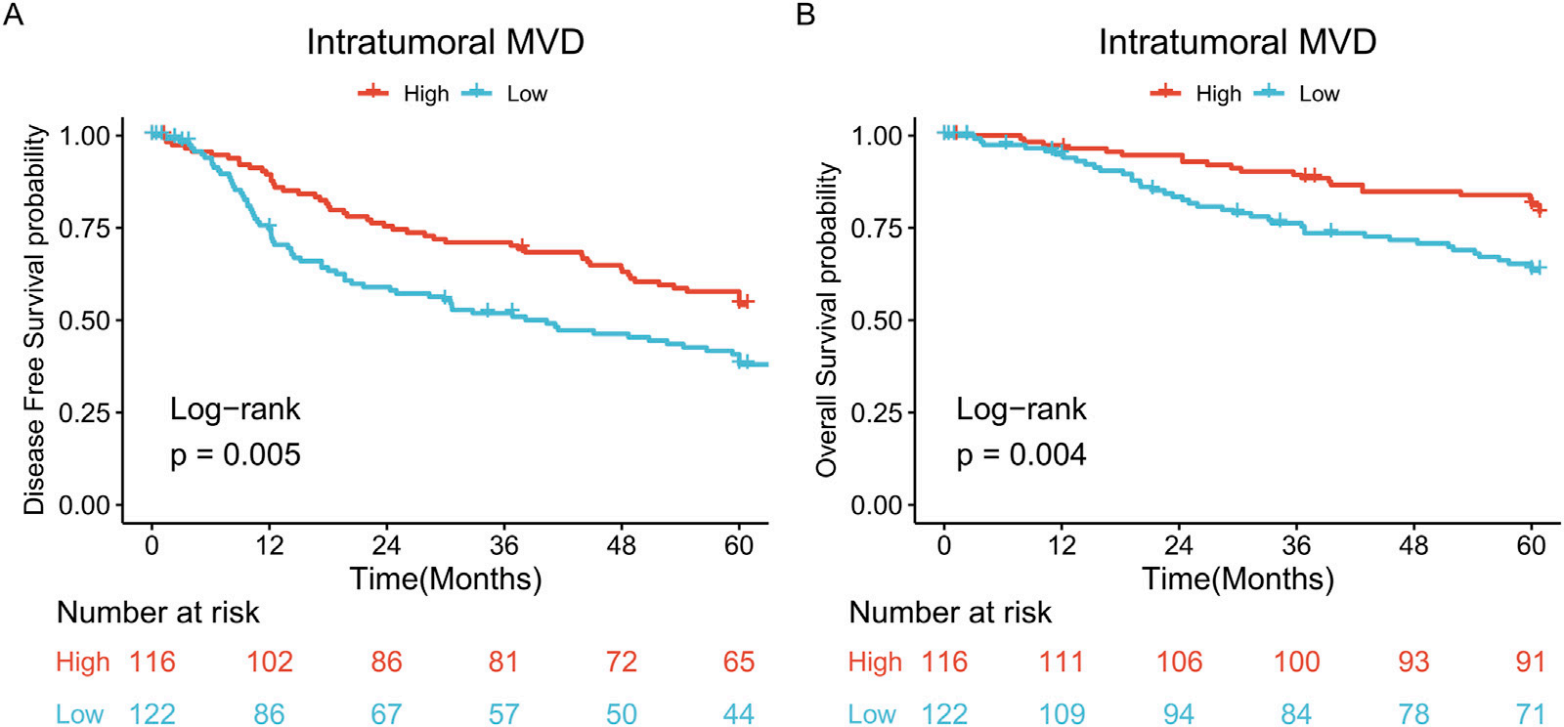
Survival analysis. **(A)** DFS curves of patients in the high and low MVD groups (log-rank *P* = 0.005); **(B)** OS curves of patients in the high and low MVD groups (log-rank *P* = 0.004).

The five-year DFS rates of patients in the low and high MVD groups were 38.0% and 54.2%, respectively, with the high MVD group showing a higher DFS rate than the low MVD group (log-rank *P* = 0.005) (Figure 3A). The five-year OS rates of patients in the low and high MVD groups were 63.4% and 81.2%, respectively, with the high MVD group showing a higher OS rate than the low MVD group (log-rank *P* = 0.004) (Figure 3B).

### Cox proportional hazards regression analysis

Cox proportional hazards regression was used to perform univariate survival analysis (including DFS and OS) for each grouping variable. The results indicated that age, sex, smoking status, tumor location, tumor size, and T stage were not associated with DFS or OS (*P* > 0.05). In contrast, lymph node metastasis status (*P* < 0.001), TNM stage (*P* < 0.001), degree of differentiation (*P* < 0.001), tumor recurrence (*P* < 0.001), and MVD (*P* = 0.005) were associated with patient survival.

Next, the positive variables were included in the construction of a multivariate Cox proportional hazards model for tumor recurrence and death after radical surgery in patients with LUAD. The results showed that the degree of differentiation (HR = 1.867, 95% CI = 1.444–2.414, *P* < 0.001), lymph node metastasis (HR = 1.779, 95% CI = 1.090–2.904, *P* = 0.021), and MVD (HR = 0.655, 95% CI = 0.456–0.941, *P* = 0.022) had statistically significant effects on DFS. This implies that poorer differentiation and the presence of lymph node metastasis increase the risk of tumor recurrence after radical surgery in patients with LUAD. In contrast, high MVD decreases the risk of tumor recurrence. Details are presented in Table 2.

**TABLE 2:** Univariate and multivariate analysis of DFS and OS.

Variables	DFS				OS			
	Univariate		Multivariate		Univariate		Multivariate	
	*P* Value	HR (95% CI)	*P* Value	HR (95% CI)	*P* Value	HR (95% CI)	*P* Value	HR (95% CI)
Age, year								
≤60*								
>60	0.599	1.100 (0.771–1.571)	—	—	0.204	1.387 (0.837–2.297)	—	—
Sex								
Female*								
Male	0.906	1.022 (0.714–1.463)	—	—	0.109	1.498 (0.914–2.456)	—	—
Smoking								
Never*								
Have	0.488	0.868 (0.582–1.294)	—	—	0.520	1.190 (0.700–2.024)	—	—
Tumor Site								
Left lung*								
Right lung	0.164	1.293 (0.900–1.858)	—	—	0.906	0.971 (0.590–1.596)	—	—
Tumor Size								
≤3 cm*								
>3 cm	0.331	0.837 (0.584–1.198)	—	—	0.847	0.952 (0.579–1.566)	—	—
T Stage								
T1-2*								
T3-4	0.722	0.924 (0.599–1.426)	—	—	0.751	1.098 (0.615–1.962)	—	—
N Stage								
N0*								
N1-3	**<0.001**	2.949 (2.045–4.251)	**0.021**	1.779 (1.090–2.904)	**<0.001**	3.683 (2.148–6.317)	0.808	1.099 (0.513–2.354)
TNM Stage	**<0.001**	1.704 (1.358–2.138)	0.172	1.242 (0.910–1.695)	**<0.001**	1.896 (1.360–2.642)	0.446	1.203 (0.748–1.937)
Stage I								
Stage II								
Stage III								
Tumor Differentiation	**<0.001**	2.142 (1.668–2.750)	**<0.001**	1.867 (1.444–2.414)	**<0.001**	4.041 (2.536–6.438)	**<0.001**	3.053 (1.803–5.171)
Well								
Moderately								
Poorly								
Tumor Recurrence								
No*								
Yes	—	—	—	—	**<0.001**	24.840 (7.780–79.310)	**<0.001**	14.275 (4.389–46.432)
MVD Grouping								
Low*								
High	**0.005**	0.600 (0.419–0.859)	**0.022**	0.655 (0.456–0.941)	**0.005**	0.474 (0.282–0.796)	0.188	0.702 (0.415–1.189)

CI, confidence interval; DFS, disease-free survival; HR, hazard ratio; MVD, microvascular density; OS, Overall survival.

Note: * As control group; Significant *P* values are presented in bold.

## Discussion

Intratumoral blood vessels provide oxygen and nutrients to tumor cells; hence, angiogenesis is thought to play an important role in tumor development. However, the processes and mechanisms underlying tumor neovascularization are complex. They may include sprouting angiogenesis, intussusceptive angiogenesis, vasculogenesis, recruitment of endothelial progenitor cells, vascular mimicry, and transdifferentiation of cancer stem cells [18]. Studies identifying various aspects of tumor angiogenesis, especially biomarkers targeting tumor angiogenesis, can help us better understand its molecular mechanisms and discover targets for designing effective anti-tumor therapies [19]. The selection of appropriate tumor vascular markers for MVD counting enables quantitative morphological analysis of the tumor vasculature and reflects the extent of tumor angiogenesis [8]. CD34, as a powerful pan-endothelial cell marker, is widely expressed in mature vascular endothelial cells and has garnered extensive interest among clinicians in clinical practice, especially in the fields of cardiovascular and cerebrovascular diseases and cancer [20]. This was the starting point of our study, in which the abundance of microvessels within LUAD tumors was quantified using CD34-MVD, and the relationship between CD34-MVD, clinicopathological characteristics, and patient prognosis was analyzed.

Our findings revealed that CD34-MVD was higher in patients with tumor size ≤3 cm than in those with tumor size >3 cm, and higher in patients without lymph node metastasis than in those with lymph node metastasis. This result reflects the fact that in LUAD, CD34 positive microvessels are more abundant in the early tumor microenvironment, which then decreases in abundance as the tumor progresses (tumor enlargement and lymph node metastasis). Our results are similar to those of Carlini et al. [21] who found that intratumoral CD34-MVD was lower in patients with stage T2 NSCLC and higher in those with stage T1 NSCLC. We analyzed two possible reasons for this result. First, CD34 positive microvessels in lung cancer tumor tissues as a differentiated microvessels tpye, which was more likely to display intratumoral mature blood vessels and peritumoral normal blood vessels [22]. Second, CD34-MVD was also shown to be associated with intratumoral necrosis, resulting in uneven oxygen distribution [16]. Thus, we hypothesized that, as the tumor increased in size, there was a decrease in mature differentiated blood vessels, an increase in intratumoral necrosis, and the occurrence of tumor hypoxia, which in turn promoted the metastasis of lung cancer to the lymph nodes. Therefore, a reduction in CD34 positive microvessels may be associated with tumor development in LUAD.

According to the survival analysis conducted in this study, patients with high CD34-MVD showed better prognosis for both DFS and OS. High CD34-MVD reduced the risk of tumor recurrence in a multivariate Cox proportional hazards regression model of postoperative tumor recurrence. Since we previously found that the decrease in CD34-MVD was associated with tumor invasion events (tumor enlargement and lymph node metastasis), thus explaining the favorable prognosis of patients with high CD34-MVD and the poor prognosis of those with low CD34-MVD. This detrimental effect of reduced CD34-MVD on prognosis has been found not only in NSCLC [16] but also in other malignancies, such as kidney cancer [23], bladder cancer [24], ovarian cancer [25], intrahepatic cholangiocarcinoma [26], and hepatocellular carcinoma [27]. Therefore, we suggest that CD34-MVD should be routinely detected in patients with lung adenocarcinoma after operation, and the patients with low CD34-MVD should be examined and evaluated for tumor recurrence more frequently during the follow-up period.

Antiangiogenic therapy, such as bevacizumab, can inhibit tumor angiogenesis and normalize tumor blood vessels. Hence, this is currently an important approach for antitumor drug therapy [28]. Some studies have found that antiangiogenic therapy, such as chemotherapy plus bevacizumab [22]or anlotinib alone [29], is more effective in patients with more undifferentiated (CD31+/CD34-) MVD in advanced NSCLC. CD34 is a marker of mature microvessels in differentiated tumors, and we found that a high abundance of CD34 positive microvessels reduced tumor invasion and improved patient prognosis. This suggests that increasing CD34 positive microvessels may be a novel approach to antitumor therapy.

Previous studies on the correlation between CD34-MVD and lung cancer have yielded mixed results, which may have been due to the following reasons. First, different MVD evaluation and counting methods were employed. Tanaka et al. [12], Kyuichi et al. [14] and Bing et al. [15] used the hotspot method in their studies, in which several areas with the densest concentration of microvessels were selected at low power and counted at high power. The average value was used as the final MVD value. In contrast, Donnem et al. [13] and Pomme et al. [16] used the microarray construction method in their studies, where MVD was defined as the number of microvessels found within a microarray core (0.6 mm in diameter). Second, different grouping criteria were used for MVD. Tanaka et al. [12] defined the high and low MVD groups using the median as the cutoff. The MVD values in the study by Donnem et al. [13] were based on IHC scoring. Kadota et al. [14] defined the high and low MVD groups by referring to the results of other previous studies. Pomme et al. [16] directly defined 65/mm^2^ as the cutoff for high and low MVD (the authors did not justify this definition). Bing et al. [15] did not divide MVD into high and low groups. Since the Weidner correction method (i.e., the hot spot method) was first proposed, it has gradually gained popularity as the most classic and widely adopted method for MVD measurement owing to its simplicity and rapidity. However, with advances in artificial intelligence and algorithms, MVD measurements assisted by computer imaging technology are expected to become a reliable indicator for evaluating therapeutic efficacy in the future [30, 31]. Based on the above, we can see that there is as yet no unified standard for MVD counting and grouping protocols, and the standardization and automation of its application should be the direction of future research.

In conclusion, our study demonstrated that a high abundance of intratumoral CD34 positive microvessels in LUAD may reduce tumor invasion, whereas a decrease in CD34 positive microvessels may be associated with tumor progression. CD34-MVD is an independent risk factor for postoperative tumor recurrence in patients with LUAD and can serve as a prognostic indicator for such patients.

## Data Availability

The original contributions presented in the study are included in the article/Supplementary Material, further inquiries can be directed to the corresponding author.
